# Supplementary material for: Residue analysis evidence for wine enriched with vanilla consumed in Jerusalem on the eve of the Babylonian destruction in 586 BCE
Source: PLoS One. 2022 Mar 29;17(3):e0266085. doi: 10.1371/journal.pone.0266085 (PMC8963535; doi:10.1371/journal.pone.0266085)
Supplement: S1 Fig — This figure was produced by the Computational Archaeology Laboratory at the Institute of Archaeology, Tel-Aviv University. (PDF) [file pone.0266085.s001.pdf]

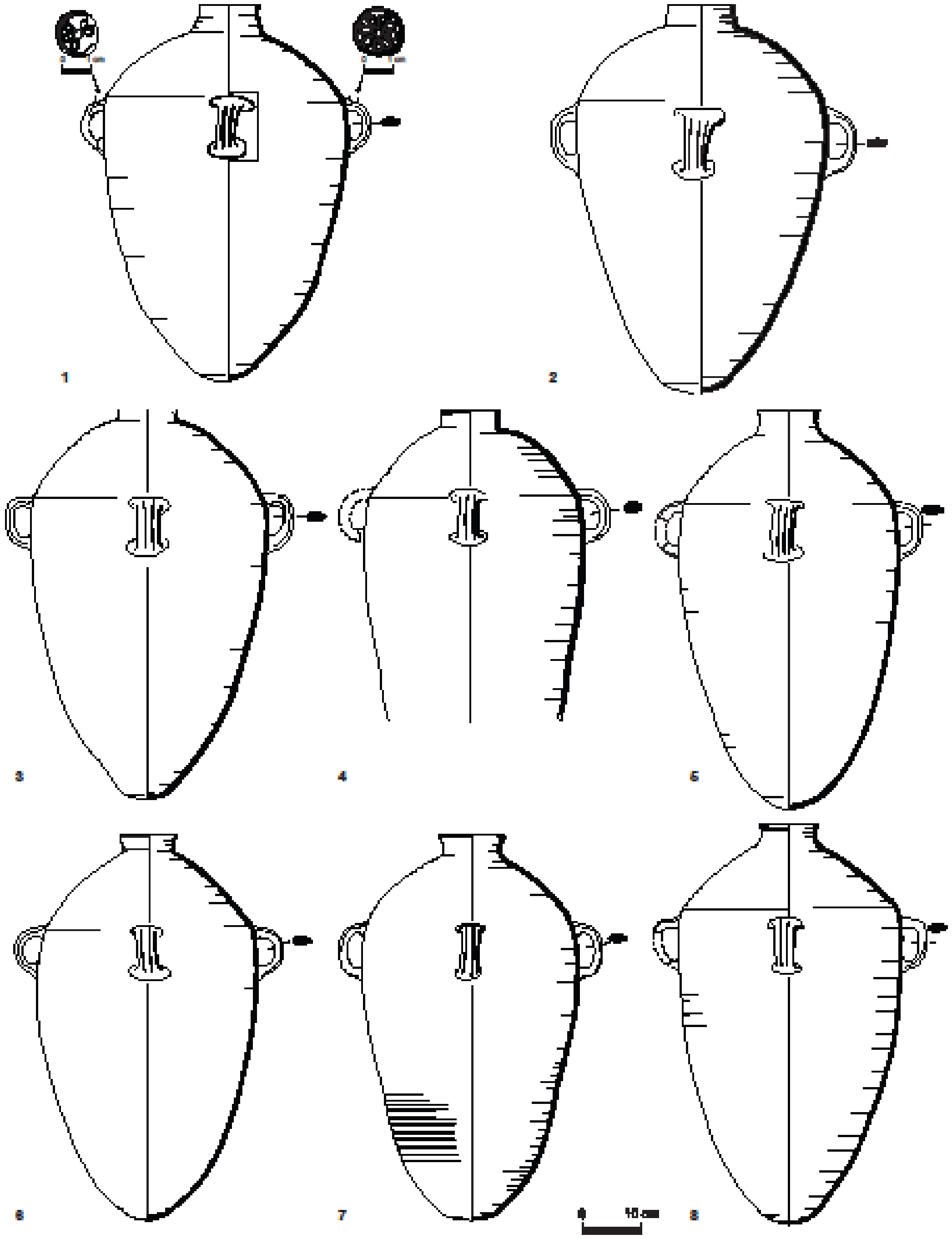

S1 Fig. A plate with the drawing of all storage jars found in Room C, Building 100, and included in this study (note that the Pithos mentioned in the text was not drawn). This figure was produced by the Computational Archaeology Laboratory at the Institute of Archaeology, Tel-Aviv University.
