# Supplementary material for: Residue analysis evidence for wine enriched with vanilla consumed in Jerusalem on the eve of the Babylonian destruction in 586 BCE
Source: PLoS One. 2022 Mar 29;17(3):e0266085. doi: 10.1371/journal.pone.0266085 (PMC8963535; doi:10.1371/journal.pone.0266085)
Supplement: S2 Fig — The pottery was digitally scanned by Ortal Harush and Argita Gyerman-Levanon. (PDF) [file pone.0266085.s002.pdf]

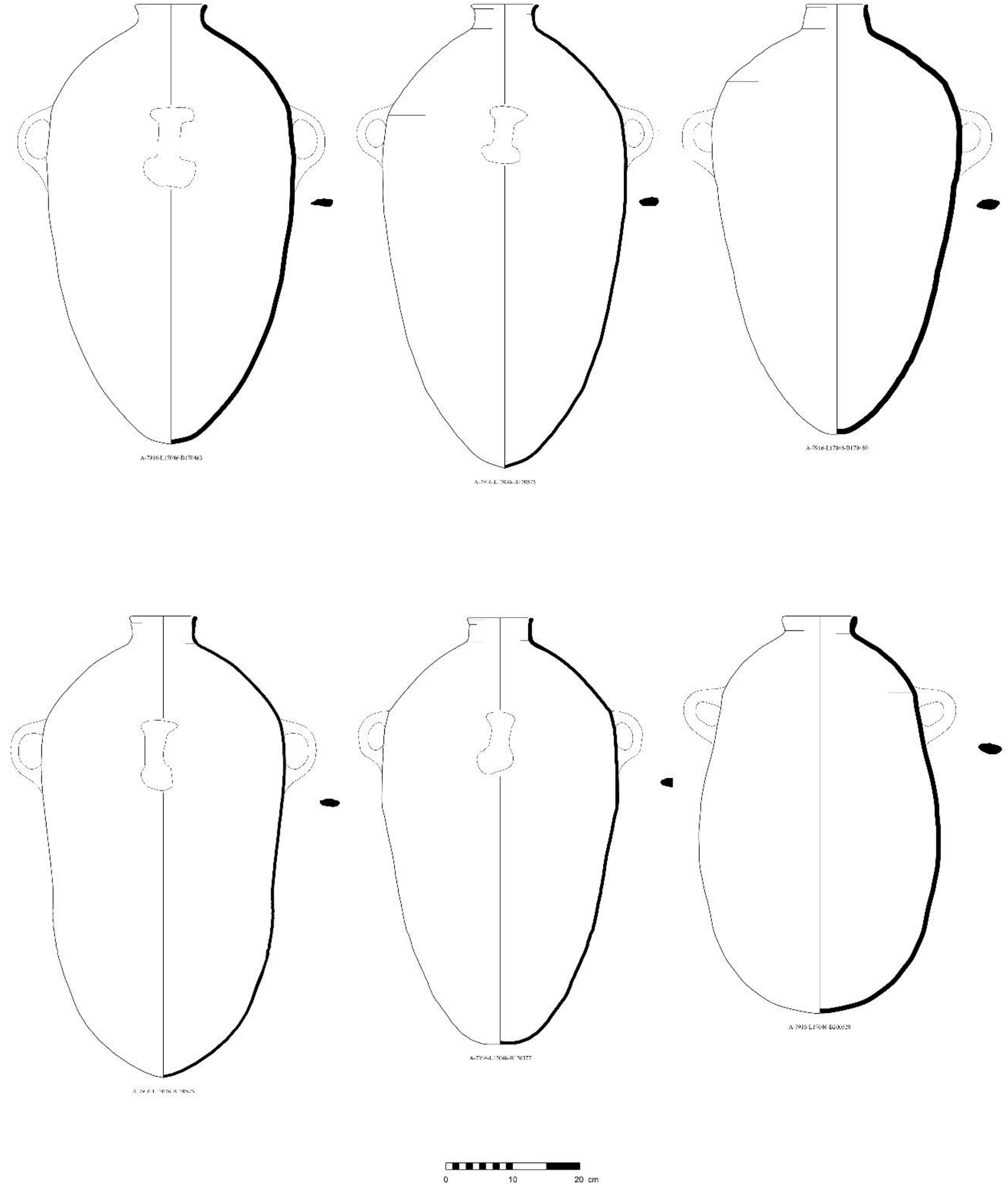

S2 Fig. A plate with the drawing of all storage jars found in Structure 17049 and included in this study. The pottery was digitally scanned by Ortal Harush and Argita Gyerman-Levanon.
