# Supplementary material for: Residue analysis evidence for wine enriched with vanilla consumed in Jerusalem on the eve of the Babylonian destruction in 586 BCE
Source: PLoS One. 2022 Mar 29;17(3):e0266085. doi: 10.1371/journal.pone.0266085 (PMC8963535; doi:10.1371/journal.pone.0266085)

GC-MS Results

Organic molecules reported are those which appeared in the vessels, while they are absent from the control and blank samples. Many molecules extracted by the TLE phase are trimethylsilylated (TMS) due to the derivatization step by silylation with BSTFA and TMC (see above, *Total Lipid Extraction*). Molecules extracted by the WM phase are butylated due to the extraction method that includes the treatment with butanol (see above, *Wine Markers Extraction*). This treatment led to different levels of butylation, manifested as multiple appearances in the chromatogram, with different numbers of butyl groups attached (butyl-, dibutyl-, tributyl-, etc.). In some rare cases the molecule couldn't be unequivocally identified, though the MS analysis pointed to a specific parent molecule that underwent some transformation. In these cases the molecule is indicated as a derivative (der.) of the parent compound in the chromatogram.

List of Abbreviations:

TMS = trimethylsilyl

P = plasticizer

C = contamination

U.I. = unidentified

.

S3 Fig. GC chromatograms of storage jars and pithoi found in Room C and included in this study.

Vessel 21748/5: sample 21753: TLE

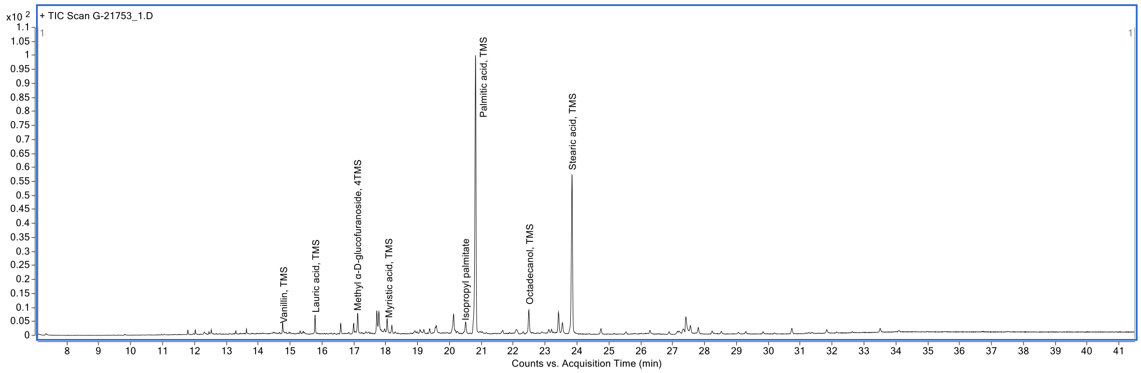

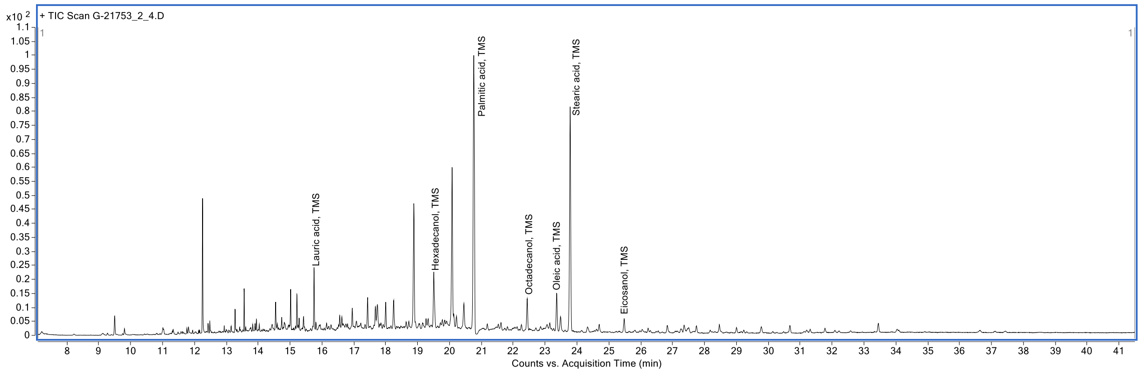

Vessel 21748/5: sample 21753: WM

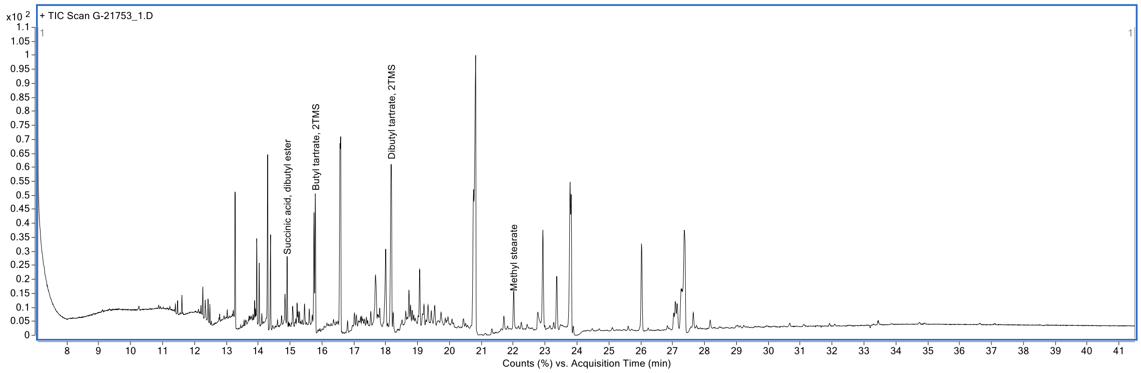

Vessel 21748/3: sample 21748_B: TLE

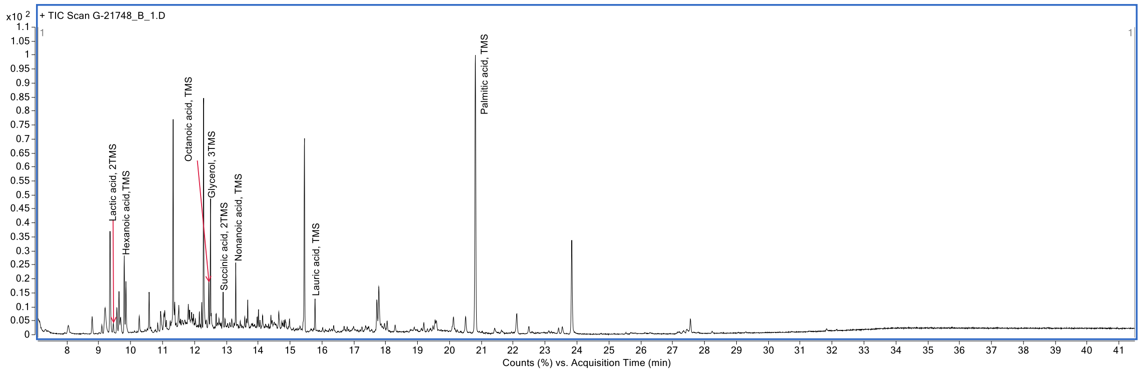

Vessel 21748/3: sample 21748_B: WM

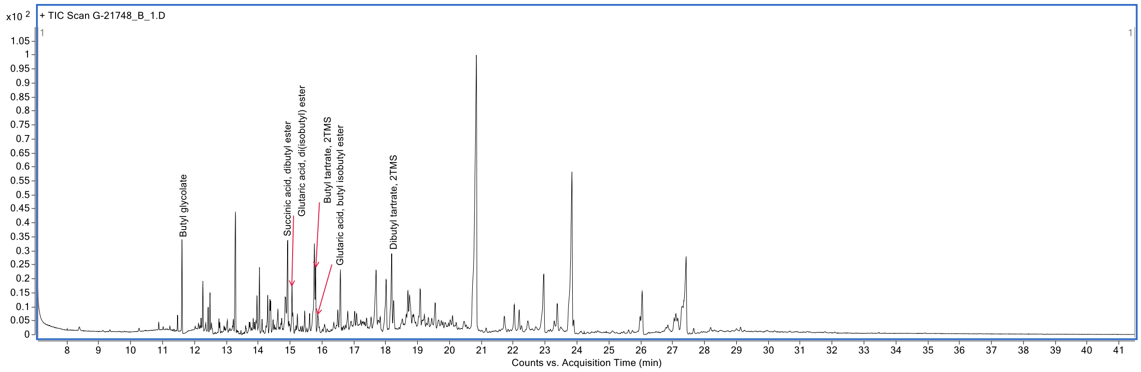

Vessel 21748/1: sample 21748_A: TLE

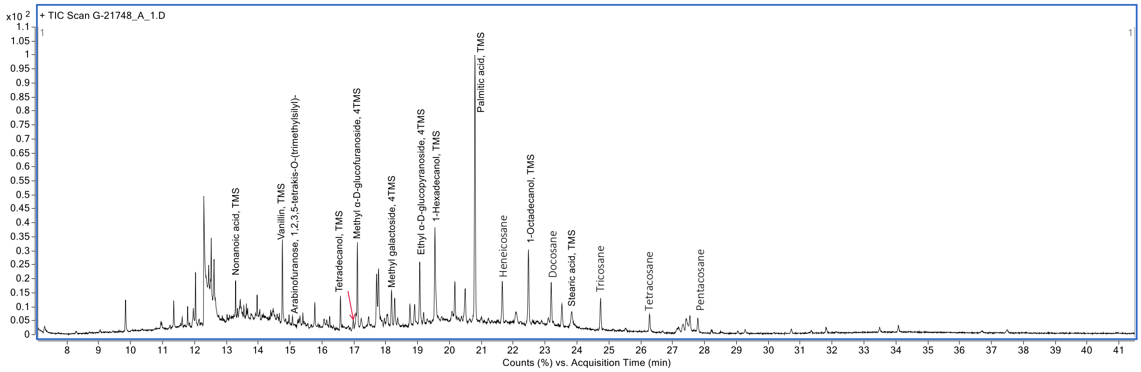

Vessel 21748/1: sample 21748_A: WM

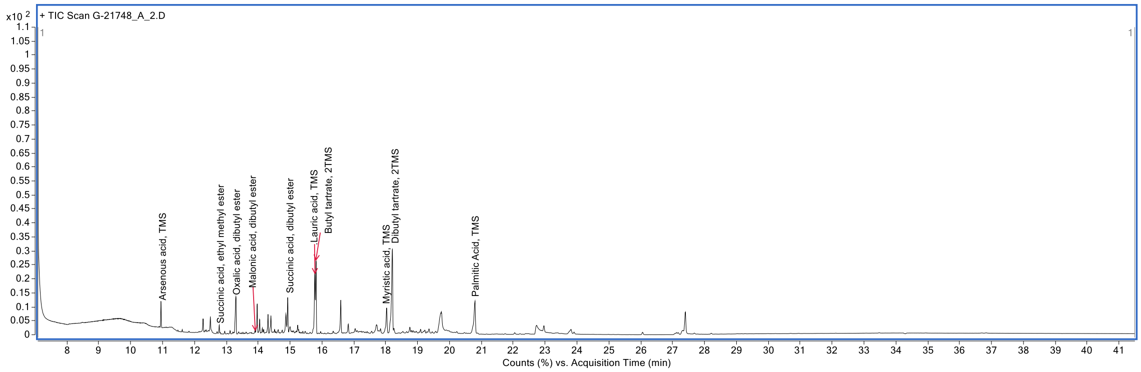

Vessel 21748/1: sample 21755: TLE

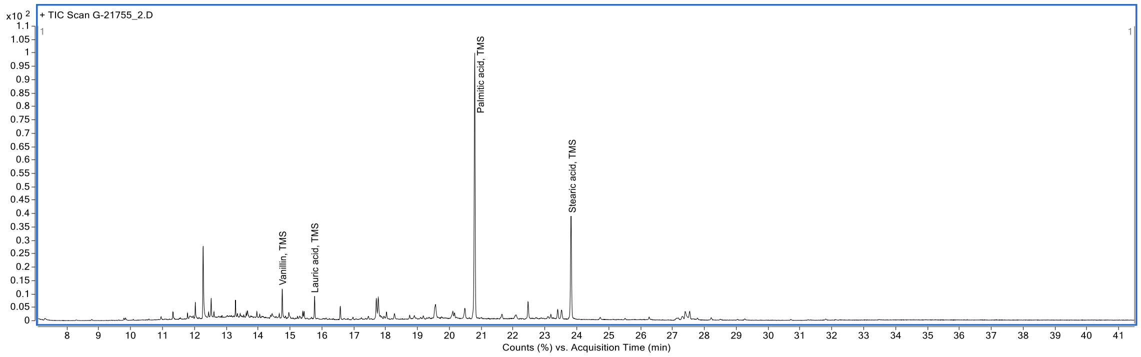

Vessel 21748/1: sample 21755: WM

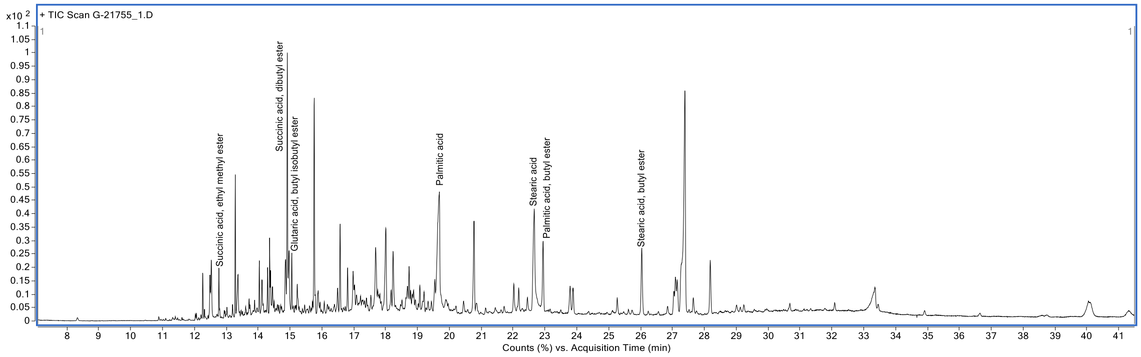

Vessel 21674/2: sample 21674: TLE

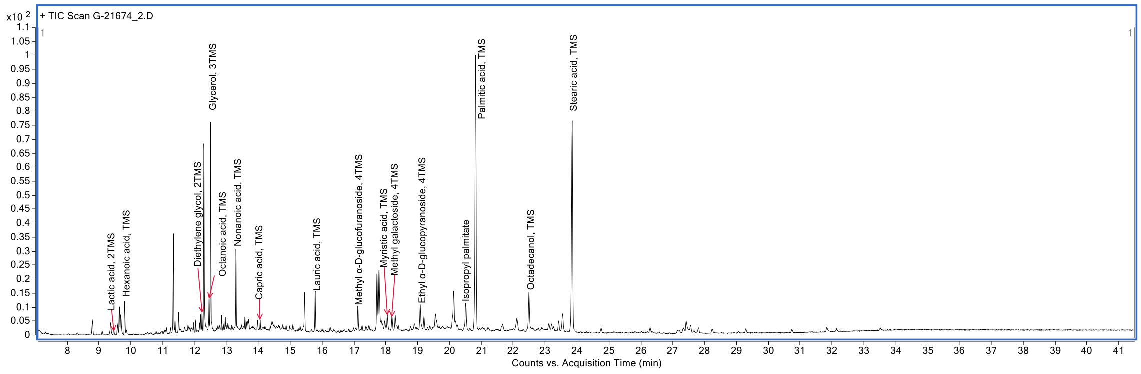

Vessel 21674/2: sample 21674: WM

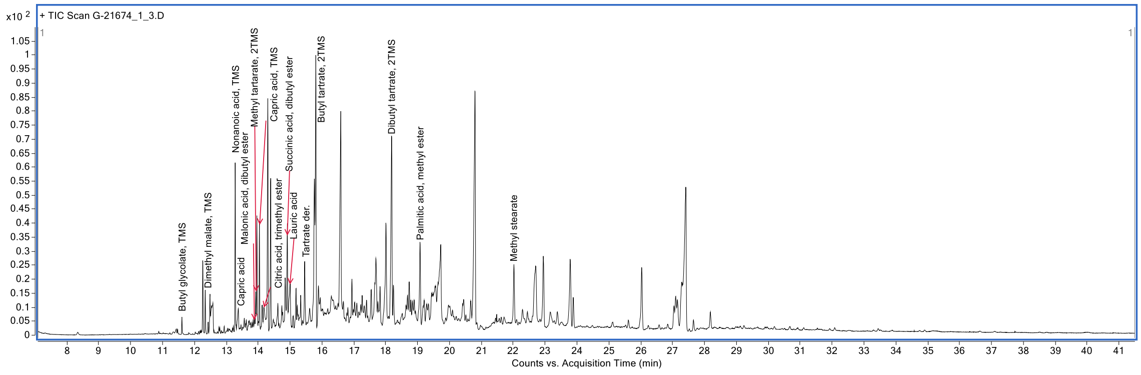

Vessel 21748/4: sample 21687: TLE

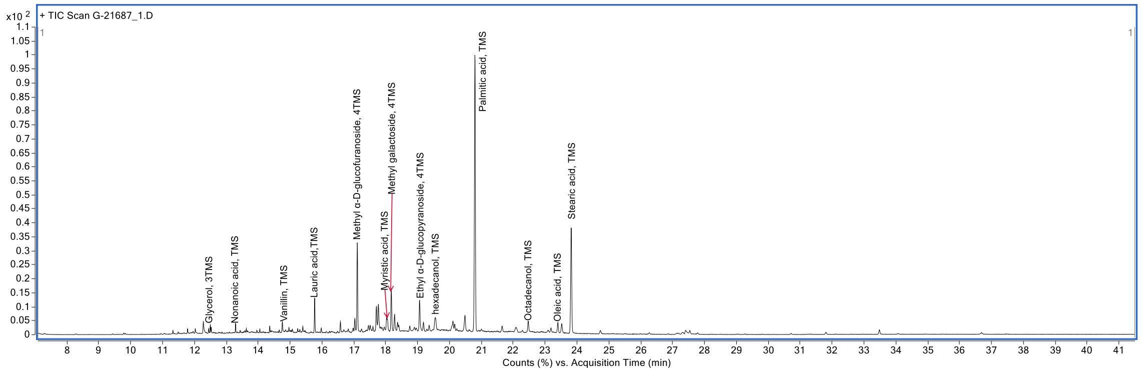

Vessel 21748/4: sample 21687: WM

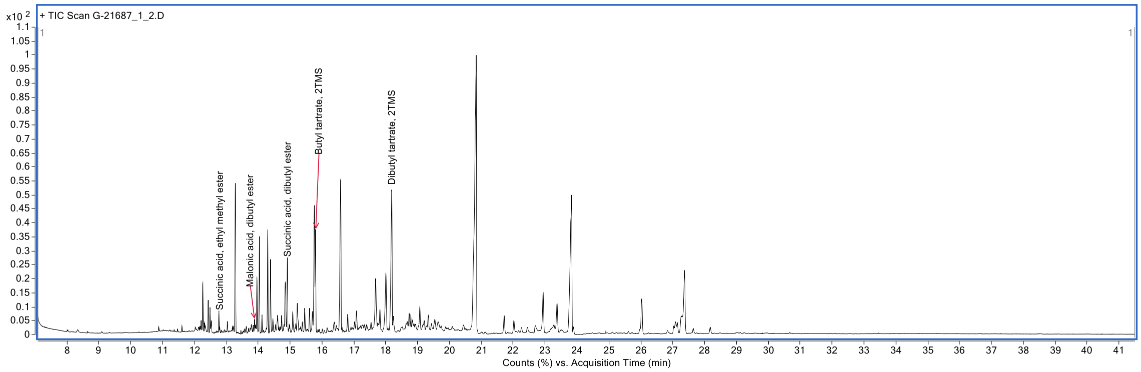

Vessel 21046/1: sample 21807 (pethos): TLE

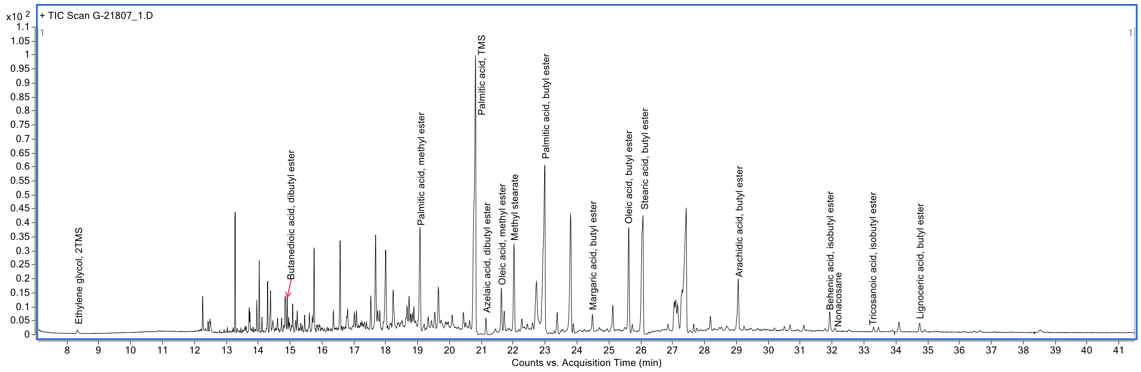

Vessel 21046/1: sample 21807 (pethos): WM

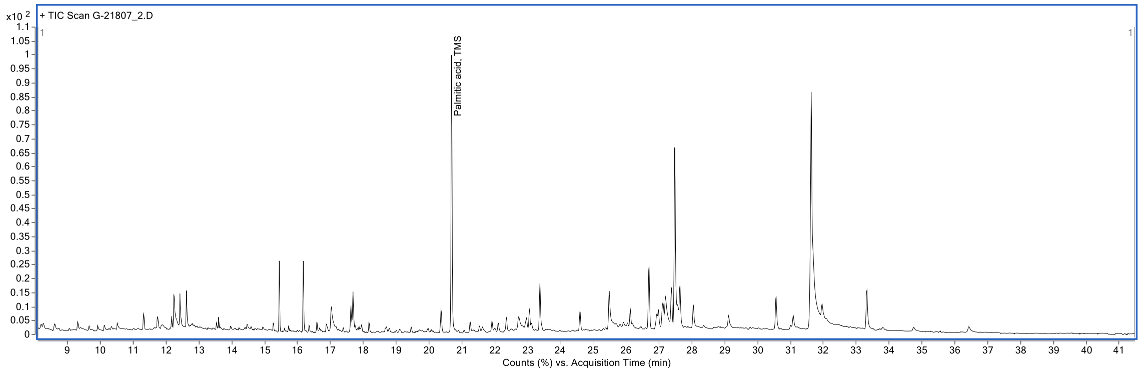

Vessel 21834/1: sample 21834: TLE

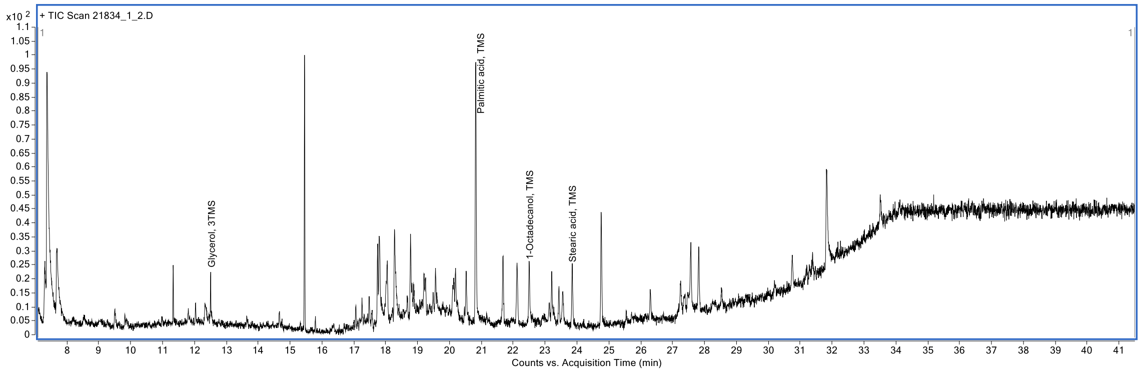

Vessel 21834/1: sample 21834: WM

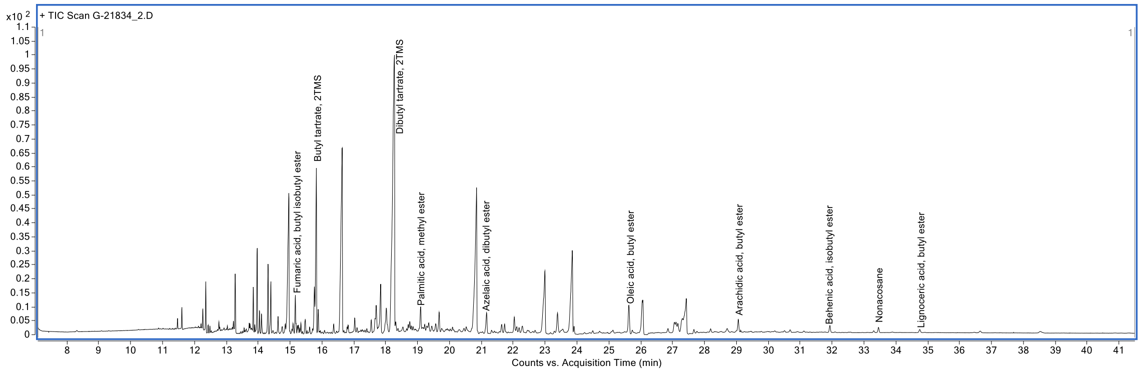

Supplement: S3 Fig — (PDF) [file pone.0266085.s003.pdf]
