# Supplementary material for: Residue analysis evidence for wine enriched with vanilla consumed in Jerusalem on the eve of the Babylonian destruction in 586 BCE
Source: PLoS One. 2022 Mar 29;17(3):e0266085. doi: 10.1371/journal.pone.0266085 (PMC8963535; doi:10.1371/journal.pone.0266085)

S4 Fig. GC chromatograms of storage jars found in Building 17049 and included in this study

Vessel 170483: sample 170430: TLE

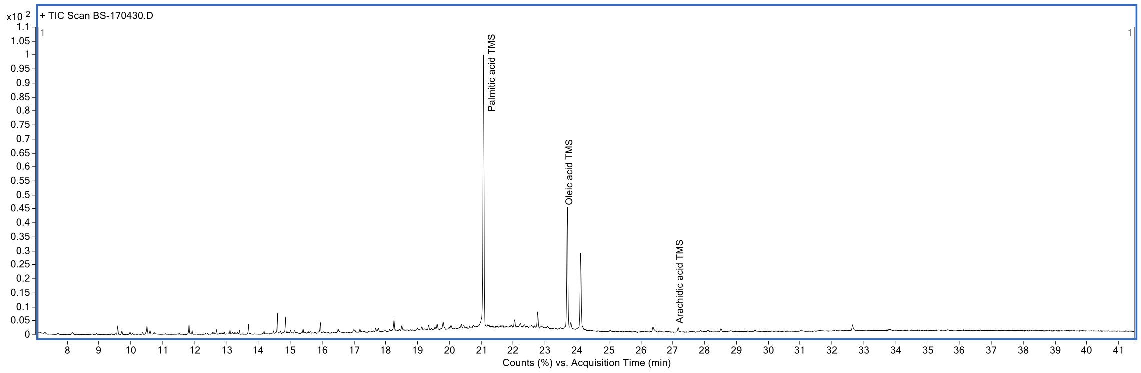

Vessel 170483: sample 170430: WM

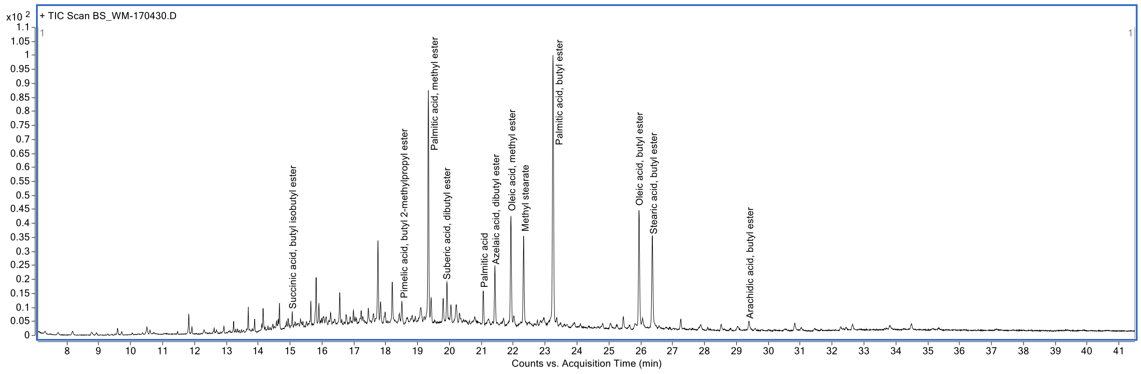

Vessel 170483: sample 170463_II: TLE

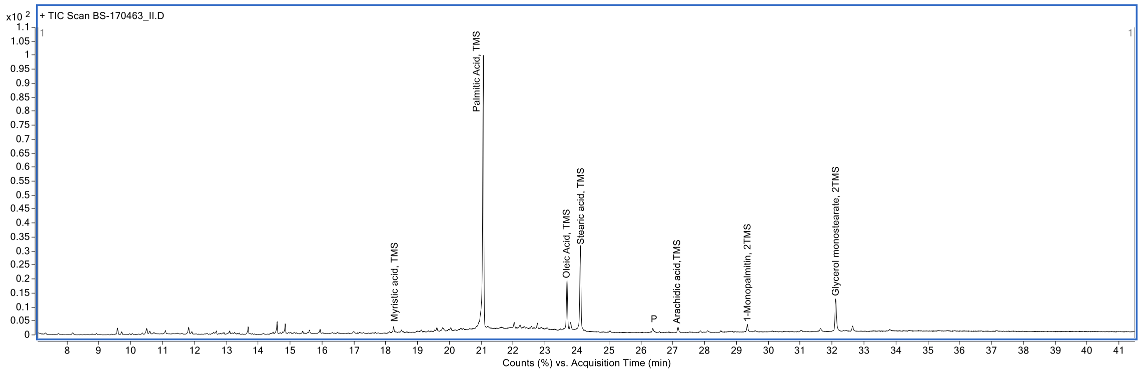

Vessel 170483: sample 170463_II: WM

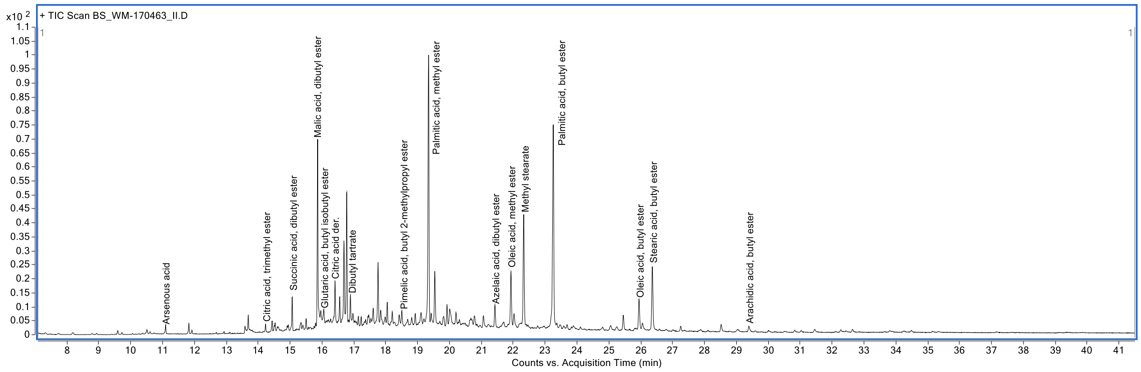

Vessel 170483: sample 170483_I: TLE

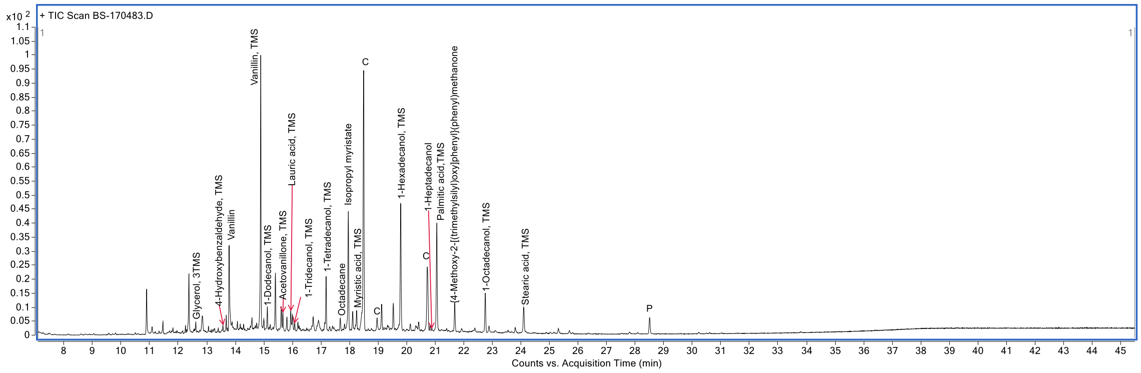

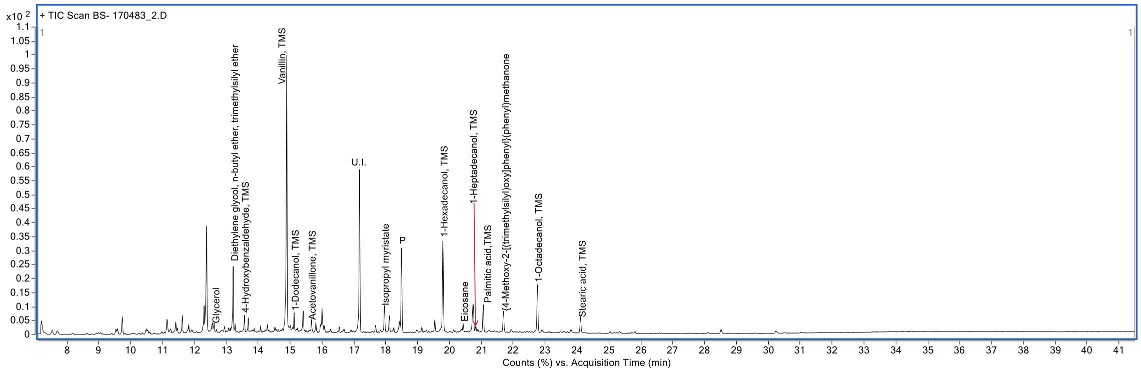

Vessel 170483: sample 170483_I: WM

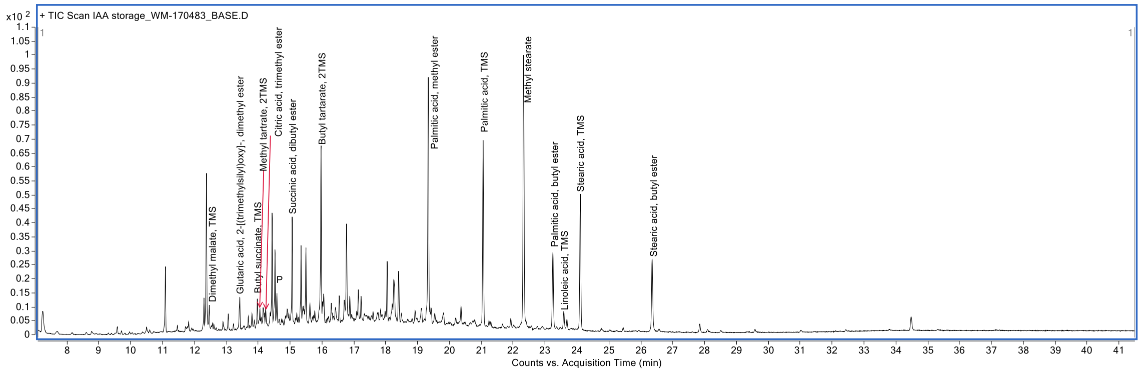

Vessel 170483: sample 170483_II: WM (TLE not analyzed)

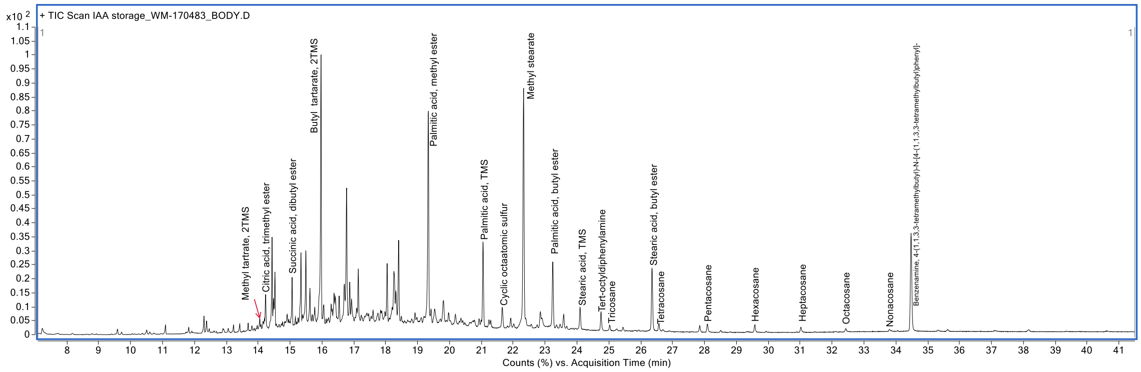

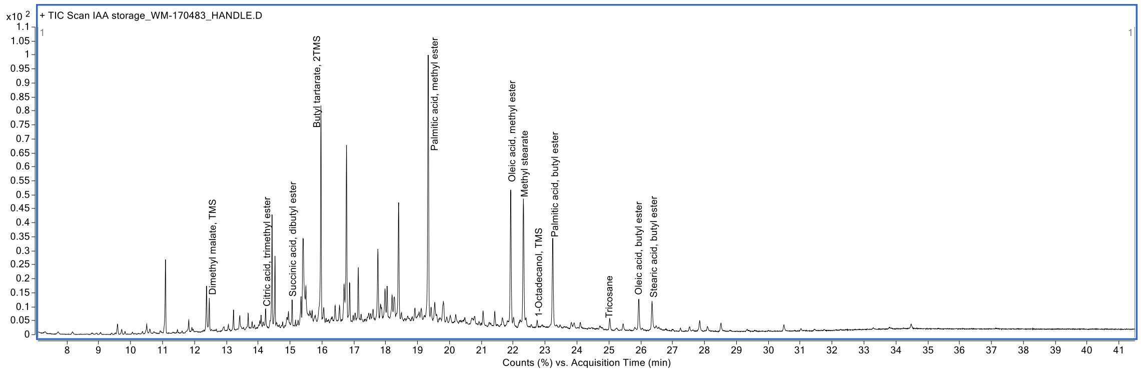

Vessel 170571: sample 170571_I: TLE

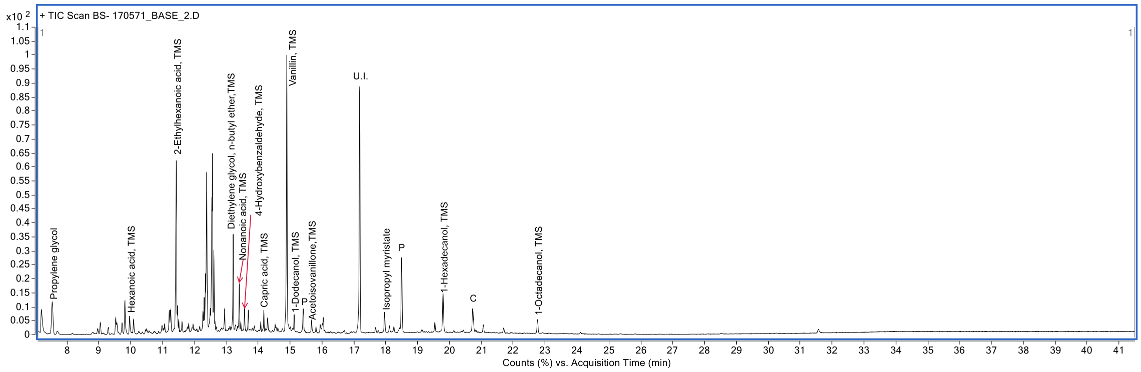

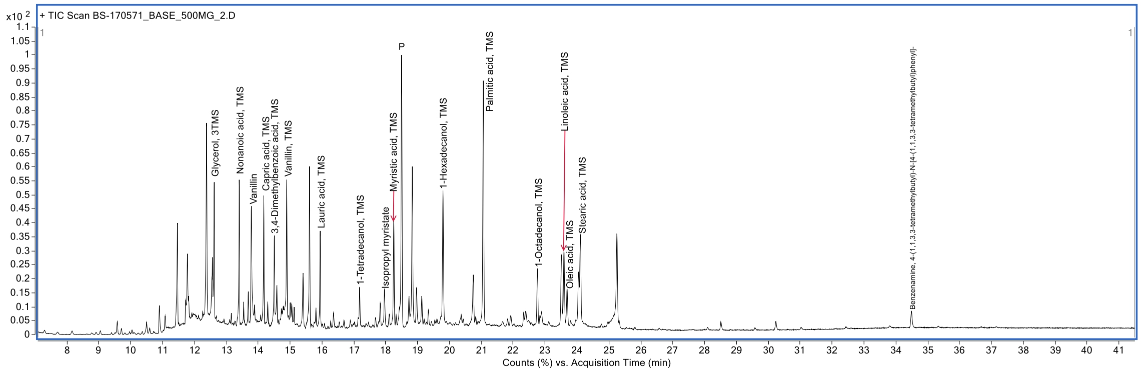

Vessel 170571: sample 170571_I: WM

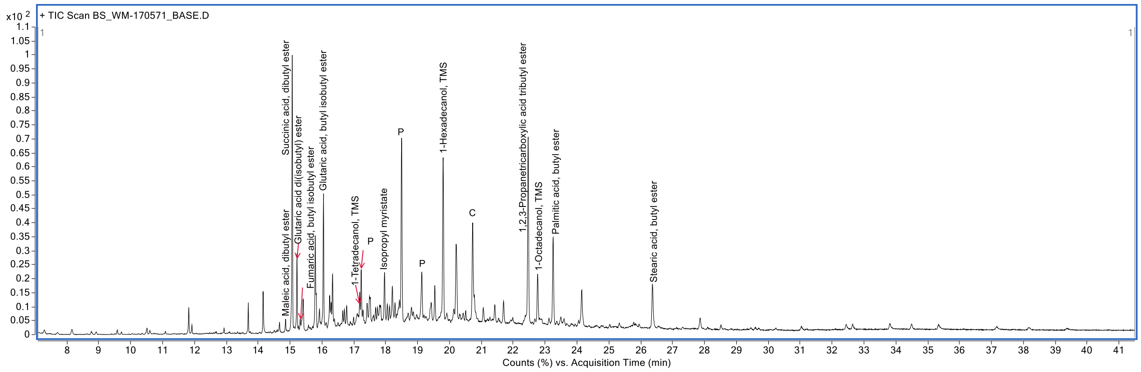

Vessel 170571: sample 170571_II: TLE

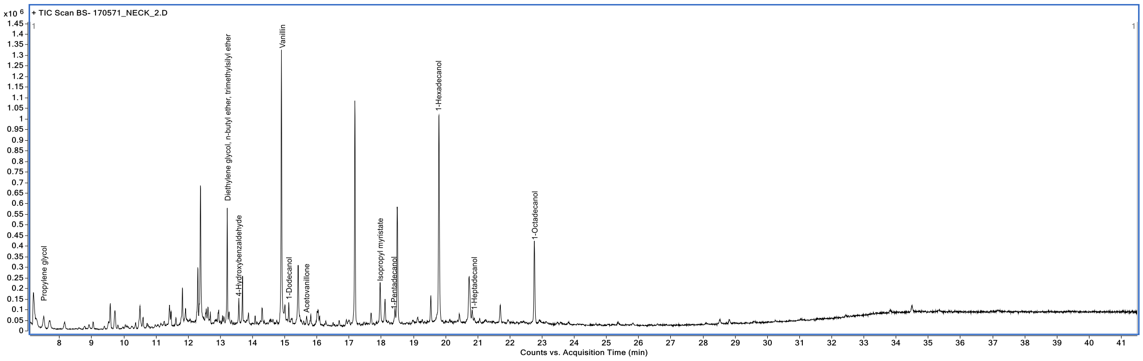

Vessel 170571: sample 170571_II: WM

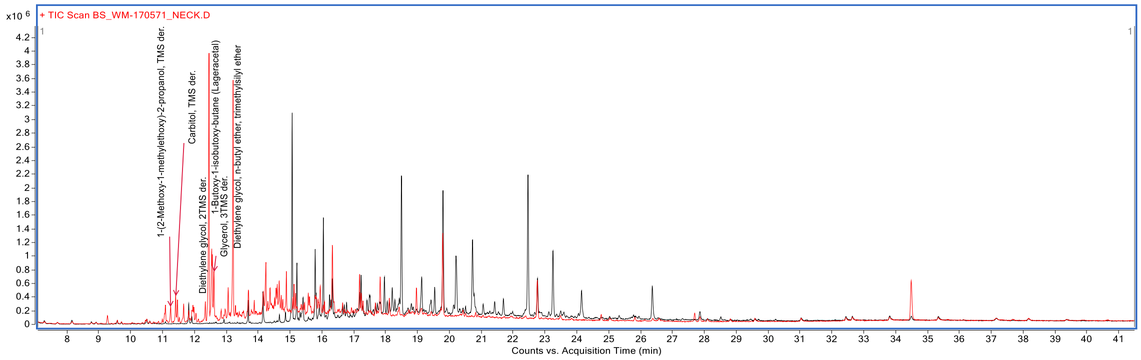

Vessel 170463_I: sample 170463_I: TLE

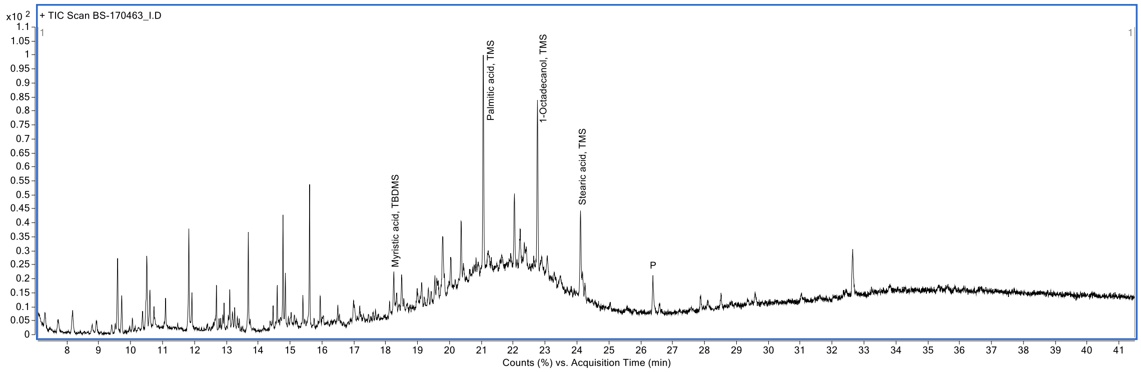

Vessel 170463_I: sample 170463_I: WM

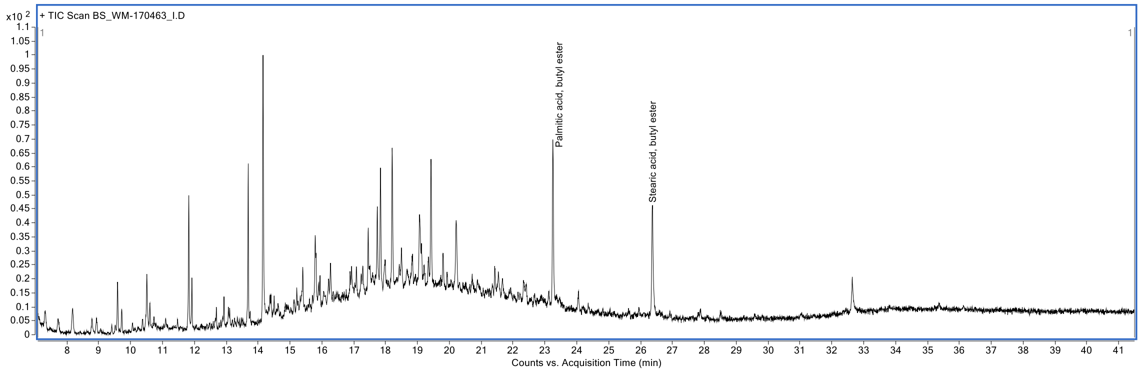

Vessel 170577/190142: sample 170577: TLE (WM not analyzed)

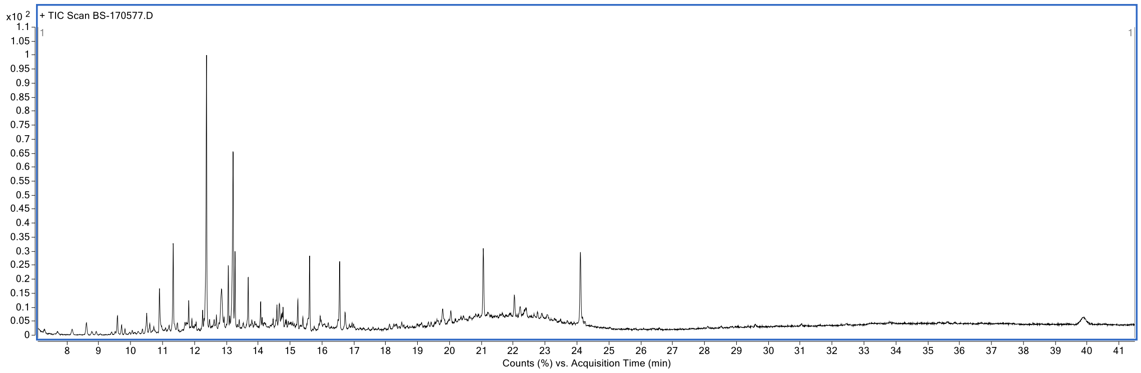

Vessel 170577/190142: sample 190142: TLE (WM not analyzed)

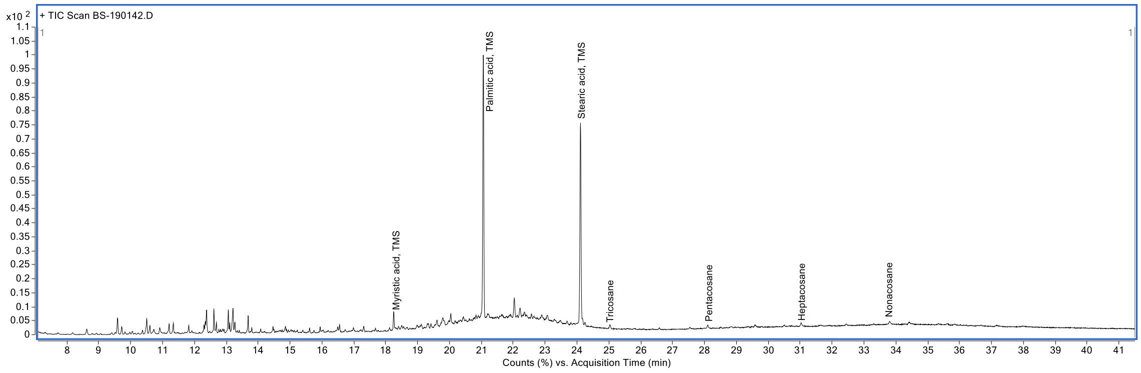

Vessel 170575: sample 170575: TLE (WM not analyzed)

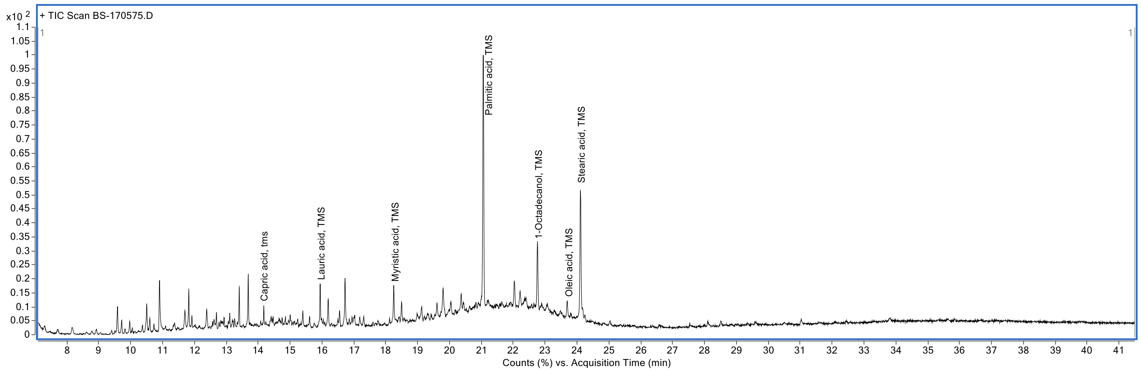

Vessel 170580: sample 170580: TLE

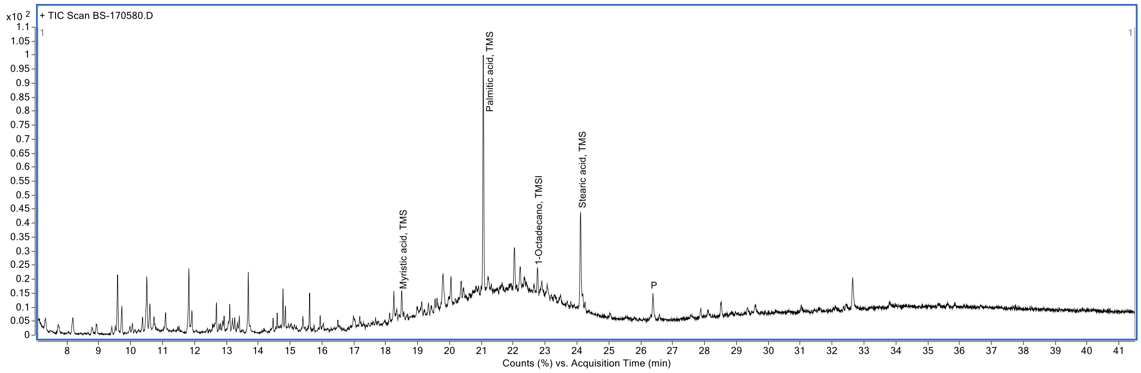

Vessel 170580: sample 170580: WM

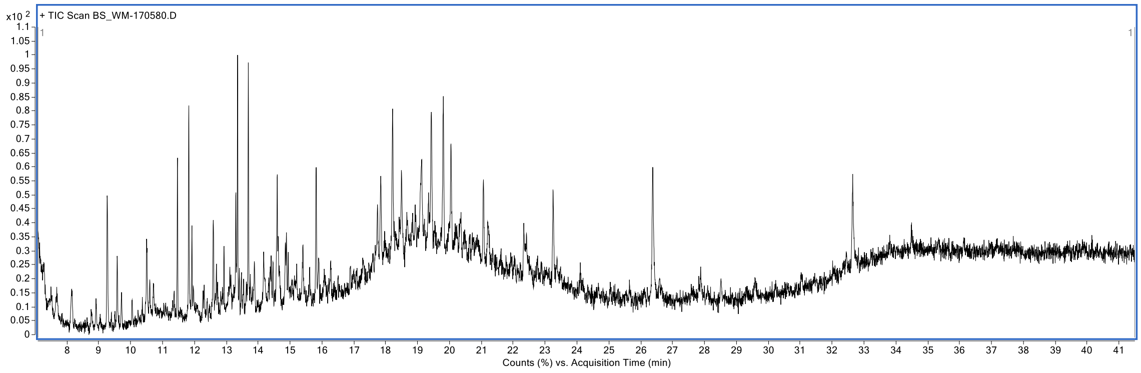

Vessel 170581: sample 170581: TLE (WM not analyzed)

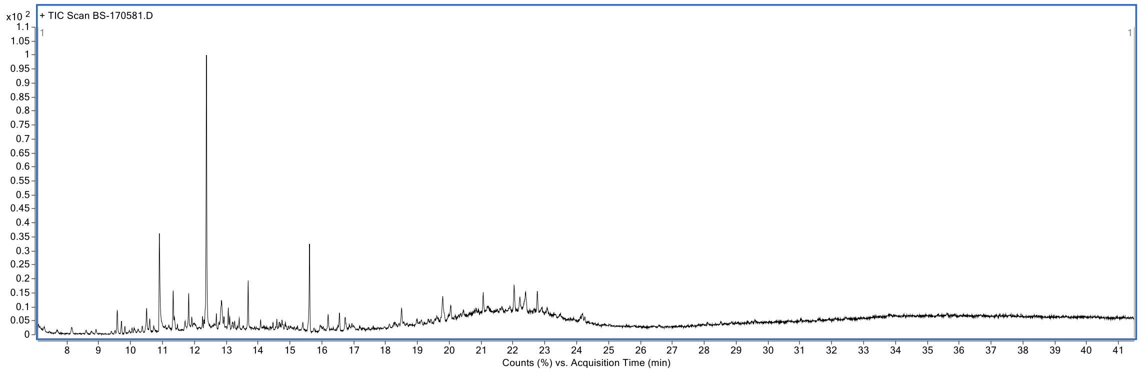

Vessel 171029: sample 171029: TLE (WM not analyzed)

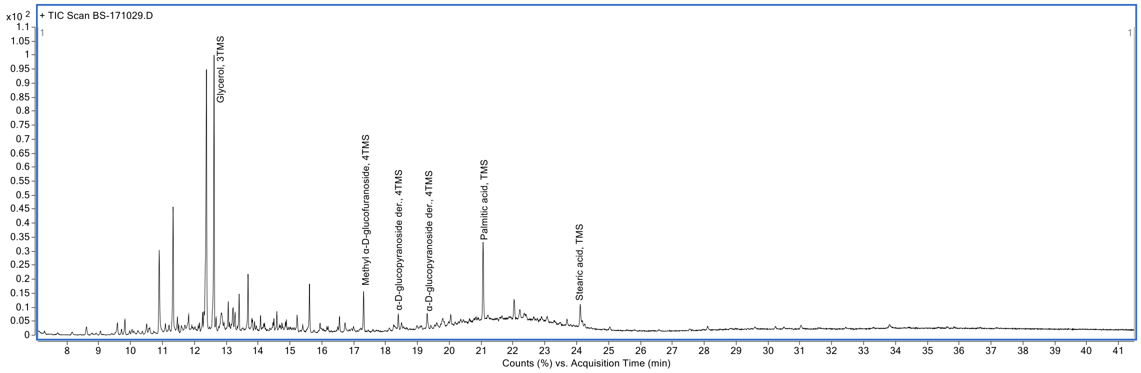

Vessel 190140/1: sample 190140: TLE (WM not analyzed)

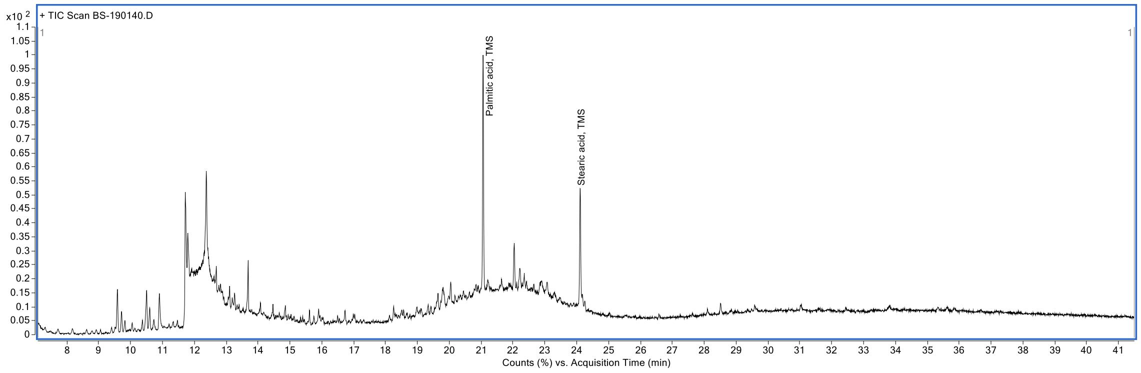

Vessel 190140/1: sample 190141: TLE (WM not analyzed)

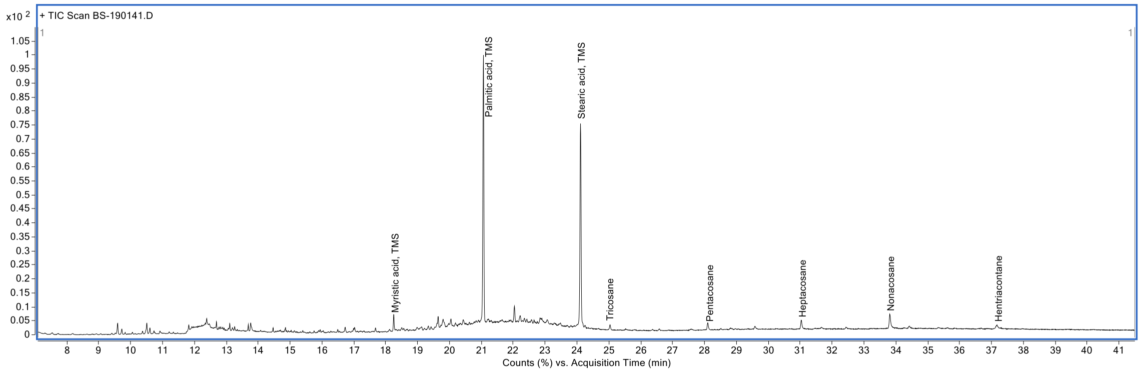

Vessel 190143: sample 190143: TLE (WM not analyzed)

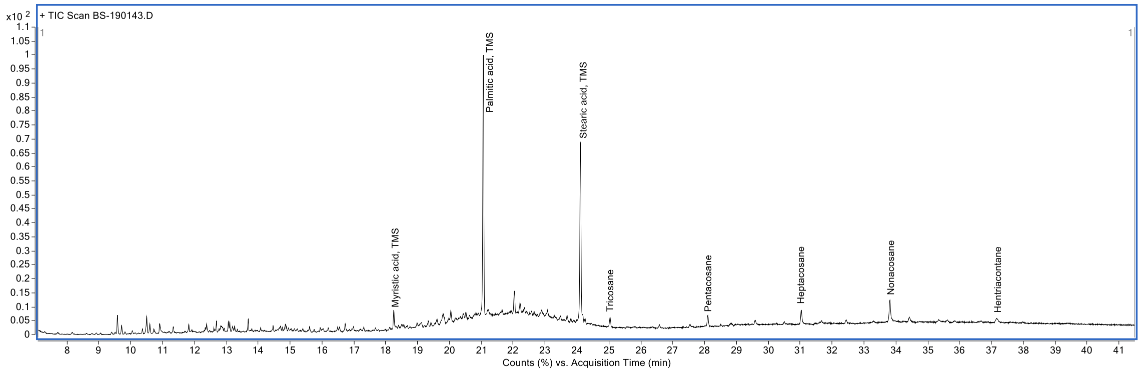

Supplement: S4 Fig — (PDF) [file pone.0266085.s004.pdf]
