# Supplementary material for: Residue analysis evidence for wine enriched with vanilla consumed in Jerusalem on the eve of the Babylonian destruction in 586 BCE
Source: PLoS One. 2022 Mar 29;17(3):e0266085. doi: 10.1371/journal.pone.0266085 (PMC8963535; doi:10.1371/journal.pone.0266085)
Supplement: S1 Table — (PDF) [file pone.0266085.s005.pdf]

**S1 Table. Givati Parking Lot Rosette storage jars from Building 100**

| | | | | |
|---|---|---|---|---|
| 1 | Rosette jar two rosette stamp impressions | 21748/1 | 1653 | Lachish Level II (ref. 1: Fig. 26.44:1); Timnah (Tel Batash) Stratum II (ref. 2: Pl. 35:1-3); Tel Ira Stratum VI (ref. 3: Fig. 6.104:12) |
| 2 | Rosette jar | 21834/1 | 1653 | As no. 1 |
| 3 | Rosette jar | 21674/3 | 1653 | Jerusalem, City of David Area G (ref. 4: Pl. 30:2); Giveati Parking Lot (ref. 5: Fig. 3.7:13-14); Lachish Level II (ref. 1: Fig. 26.44:6); En-Gedi Stratum V (ref. 6: Pl. 10:1-4); Timnah (Tel Batash) Stratum II (ref. 2: Pl. 46: 2)<br>Tel Ira Stratum VI (ref. 3: Fig. 6.91: 16, with two rosette stamp impressions, Fig. 6.92: 23 with rosette stamp impression); Tel Malhata Stratum IIIA (ref. 7: Fig. 4.73:6; and Figs. 4.162:1; 170:11 each with rosette stamp impression |
| 4 | Rosette jar | 21674/2 | 1653 | As no. 3 |
| 5 | Rosette jar with square section rim | 21748/4 | 1653 | Arad Stratum VII (ref. 8: Fig. 46:7); En-Gedi Stratum V with similar cut rim (ref. 6: Pl. 10:3); Timnah (Tel Batash) Stratum II (ref. 2: Pl. 46:1,3) |
| 6 | Rosette jar | 21674/1 | 1653 | As no. 3 |
| 7 | Rosette jar | 21748/3 | 1653 | As no. 3 |
| 8 | Rosette jar | 20851/1 | 1563 | As no. 3 |

# References


1. Zimhoni O. The Pottery of Levels III and II. In: Ussishkin D, editor. The Renewed Archaeological Excavations at Lachish (1973-1994) . Tel Aviv: Emery and Claire Yass Publications in Archaeology, Institute of Archaeology, Tel Aviv University; 2004. pp. 1789–1900.
2. Mazar A, Panitz-Cohen N. Timnah (Tel Batash) II: The Finds from the First Millennium BCE, Jerusalem: Institute of Archaeology, Hebrew University of Jerusalem; 2001.
3. Freud L. Pottery: The Iron Age. In: Freud L, Beit-Arieh I, editors. Tel ‘Ira: A Stronghold in the Biblical Negev (Monograph Series of the Institute of Archaeology of Tel Aviv University No 15). Tel Aviv: Emery and Claire Yass Publications in Archaeology, Institute of Archaeology, Tel Aviv University; 1999. pp. 189–289.
4. Shiloh Y. Excavations at the City of David I: 1978-1982: Interim Report of the First Five Seasons. Jerusalem: Institute of Archaeology, Hebrew University of Jerusalem; 1984.
5. Ben Ami D. Jerusalem: Excavations in the Tyropoeon Valley, (Giv’ati Parking Lot). Israel Antiquities Authority; 2013.
6. Yezerski I. Pottery of stratum V. In: Stern E, editor. En-Gedi Excavations I, Conducted by B Mazar and I Dunayevsky, Final Report. Jerusalem: Israel Exploration Society, Institute of Archaeology, Hebrew University of Jerusalem; 2007. pp. 86–129.
7. Freud L. The pottery of strata v–iii. Tel Malhata: A central city in the biblical Negev. Penn State University Press; 2015. pp. 153–486. doi:10.5325/j.ctv1bxh406.8
8. Singer-Avitz L. Arad: the Iron Age pottery assemblages. Tel Aviv. 2002;29: 110–214. doi:10.1179/tav.2002.2002.1.110
