# Supplementary material for: Residue analysis evidence for wine enriched with vanilla consumed in Jerusalem on the eve of the Babylonian destruction in 586 BCE
Source: PLoS One. 2022 Mar 29;17(3):e0266085. doi: 10.1371/journal.pone.0266085 (PMC8963535; doi:10.1371/journal.pone.0266085)
Supplement: S2 Table — (PDF) [file pone.0266085.s006.pdf]

S2 Table. RA results of all vessels included in this study

Arch. context	Sampling context	# Vessel	# Sample	TLE	WM
Structure 17049	IAA storage room	170483	170430	$C_{16:0}$, $C_{18:1}$, $C_{20:0}$	succinic, pimelic, suberic, azelaic, $C_{16:0}$, $C_{18:0}$, $C_{18:1}$, $C_{20:0}$
			170463_II	$C_{14:0}$, $C_{16:0}$, $C_{18:1}$, $C_{18:0}$, $C_{20:0}$, $MAG_{16:0}$, $MAG_{18:0}$	arsenous, succinic, malic, glutaric, citric, tartaric, pimelic, $C_{16:0}$, azelaic $C_{18:1}$, $C_{18:0}$, $C_{20:0}$
	In Situ		170483_I (base)	Propylene glycol, diethylene glycol, glycerol, 4-hydroxybenzaldehyde, vanillin, C_{12ol}, acetovanillone, $C_{12:0}$, C_{13ol}, C_{14ol}, C_{18}, isopropyl myristate, $C_{14:0}$, C_{16ol}, C_{20}, C_{17ol}, $C_{16:0}$, {4-Methoxy-2-[(trimethylsilyl)oxy]phenyl}(phenyl)methanone, C_{18ol}, $C_{18:0}$	dimethyl malate, glutaric, tartaric, citric, succinic, $C_{16:0}$, $C_{18:0}$, $C_{18:2}$
	IAA storage room		170483_II (body)	Not analyzed	Dimethyl malate, tartaric, citric, succinic, fumaric, glutaric, $C_{16:0}$, cyclic octaatomic sulfur, C_{18ol}, $C_{18:1}$, $C_{18:0}$, C_{23-29}, benzenamine, 4(1,1,3,3-tetramethylbutyl)-N-[4(1,1,3,3-tatramethylbutyl)phenyl]-
	In Situ	170571	170571_I (base)	Propylene glycol, $C_{6:0}$, diethylene glycol, tripopylen glycol, glycerol, $C_{9:0}$, 4-hydroxybenzaldehyde, $C_{10:0}$, Vanillin, $C_{12:0}$, $C_{14:0}$, C_{12ol}, acetovanillone, 3,4 dimethyl benzoic acid, C_{14ol}, isopropyl myristate, C_{16ol}, $C_{16:0}$, C_{17ol}, C_{18ol}, $C_{18:1; 13}$, $C_{18:1}$, $C_{18:0}$, octanamine, C_{20ol}	Maleic, succinic, oxalic, Fumaric, glutaric, trimethyl **1,2,3-propanetricarboxylate,** C_{14ol}, isopropyl myristate, C_{16ol}, **1,2,3-Propanetricarboxylic acid** C_{18ol}, $C_{16:0}$, $C_{18:0}$
	In Situ		170571_II (neck)	Propylene glycol, diethylene glycol, 4-hydroxybenzaldehyde, Vanillin, C_{12ol}, acetovanillone, isopropyl myristate, C_{15ol}, C_{16ol}, C_{17ol}, C_{18ol}	Glycerol, diethylene glycol
	IAA storage room	170463_I	170463_I	$C_{14:0}$, $C_{16:0}$, $C_{18:0}$, C_{18ol},	$C_{16:0}$, $C_{18:0}$
	IAA storage room	170577/ 190142	170577	---	Not analyzed
	In situ, wet		190142	$C_{16:0}$, $C_{18:0}$, C_{23}, C_{25}, C_{27}, C_{29}, C_{31}	Not analyzed
	IAA storage room	170575	170575	$C_{10:0}$, $C_{12:0}$, $C_{14:0}$, $C_{16:0}$, C_{18ol}, $C_{18:1}$, $C_{18:0}$	Not analyzed
		170580	170580	$C_{14:0}$, $C_{16:0}$, C_{18ol}, $C_{18:0}$	---
		170581	170581	---	Not analyzed
		171029	171029	Glycerol, sugars, $C_{16:0}$, $C_{18:0}$	Not analyzed
	In situ, wet	190140/1	190140	$C_{16:0}$, $C_{18:0}$, C_{23}, C_{25}, C_{27}, C_{29}, C_{31}	Not analyzed
			190141	$C_{14:0}$, $C_{16:0}$, $C_{18:0}$, C_{23}, C_{25}, C_{27}, C_{29}, C_{31}	Not analyzed
		190143	190143	$C_{14:0}$, $C_{16:0}$, $C_{18:0}$, C_{23}, C_{25}, C_{27}, C_{29}, C_{31}	Not analyzed

Building 100	Lab (in proximity to exposure)	21748/5	21753	$C_{12:0}$, C_{16ol}, $C_{16:0}$, C_{18ol}, $C_{18:1}$, $C_{18:0}$, C_{20ol}	Succinic, tartaric, $C_{18:0}$
		21748/3	21748_B	Lactic, $C_{6:0}$, $C_{8:0}$, glycerol, succinic, $C_{9:0}$, $C_{12:0}$, $C_{16:0}$	Butyl glycolate, succinic, glutaric, tartaric,
		21748/1	21748_A	$C_{9:0}$, vanillin, sugars, C_{14ol}, C_{16ol}, $C_{16:0}$, C_{21-25}, C_{18ol}, $C_{18:0}$	Arsenous, succinic, oxalic, malonic, $C_{12:0}$, tartaric, $C_{14:0}$, $C_{16:0}$
			21755	Vanillin, $C_{12:0}$, $C_{16:0}$, $C_{18:0}$	Succinic, glutaric, $C_{16:0}$, $C_{18:0}$
		21674/2	21674	Lactic, $C_{6:0}$, diethylen glycol, $C_{8:0}$, glycerol, $C_{9:0}$, $C_{10:0}$, $C_{12:0}$, sugars, $C_{14:0}$, $C_{16:0}$, C_{18ol}, $C_{18:0}$	Butyl glycolate, dimethyl malate, $C_{9:0}$, $C_{10:0}$, tartaric, citric, $C_{13:0}$, levoglucosan, succinic, $C_{12:0}$, $C_{16:0}$, $C_{18:0}C_{14:0}$
		21748/4	21687	Glycerol, $C_{9:0}$, vanillin, $C_{12:0}$, glycosides, $C_{14:0}$, C_{16ol}, $C_{16:0}$, C_{18ol}, $C_{18:1}$, $C_{18:0}$	malonic, succinic, tartaric
		21046/1 (pethos)	21807	$C_{16:0}$	Ethylene glycol, succinic, $C_{16:0}$, azelaic, $C_{17:0}$, $C_{18:1}$, $C_{18:0}$, $C_{20:0}$, $C_{22:0}$, C_{29}, $C_{23:0}$, $C_{24:0}$
		21834/1	21834	Glycerol, $C_{16:0}$, C_{18ol}, $C_{18:0}$	fumaric, tartaric, azelaic, $C_{16:0}$, $C_{18:1}$, $C_{18:0}$, $C_{20:0}$, $C_{22:0}$, C_{29}, $C_{24:0}$
