# Supplementary material for: Residue analysis evidence for wine enriched with vanilla consumed in Jerusalem on the eve of the Babylonian destruction in 586 BCE
Source: PLoS One. 2022 Mar 29;17(3):e0266085. doi: 10.1371/journal.pone.0266085 (PMC8963535; doi:10.1371/journal.pone.0266085)
Supplement: Attachment — Submitted filename: Response to Reviewers.docx [file pone.0266085.s008.docx]

Dear Editor,

Thank you for considering our manuscript. We highly appreciated the Reviewers comments, and corrected the manuscript accordingly. Below please find our detailed response.

Sincerely,

Ayala Amir on behalf of all other authors

1. The manuscript was adapted to PLOS ONE's style requirements.
2. Our ethics statement was moved to the Methods section of the manuscript.
3. The excavation map (figure 1) was prepared by the City of David expedition, headed by three of the authors, and the full copyright belongs to them. This map hadn’t been published in any other journal. This information was included in the ethic statement section, and the appropriate credit was included in the figure legend as well.
4. The references were adapted to PLOS ONE's style requirements. Reference number 14 has been removed as it has not yet been published and there are other references in the article that address the relevant topic in this reference.
5. The introduction and the conclusions were edited in order to make sure they fit better the body of the article (results and discussion), following the comment made by the first reviewer.
6. following the comments made by the second reviewer:

- Significant figures were added to the quantities mentioned in the methods section so two significant figures are quoted. (The quantities of reagents used in the extraction and silylation procedures are in large excess of the molecules with which they react in order to ensure complete extraction and derivatization. Therefore, there is no need for such accuracy).
- The words “an aqueous” were added to the relevant sentence.
- The word “Anhydrous” was added to the relevant sentence.
